# Bulevirtide Monotherapy Is Safe and Well Tolerated in Chronic Hepatitis Delta: An Integrated Safety Analysis of Bulevirtide Clinical Trials at Week 48

**DOI:** 10.1111/liv.16174

**Published:** 2024-12-08

**Authors:** Tarik Asselah, Pietro Lampertico, Soo Aleman, Marc Bourlière, Adrian Streinu‐Cercel, Pavel Bogomolov, Viacheslav Morozov, Tatiana Stepanova, Stefan Lazar, Dmitry Manuilov, Renee‐Claude Mercier, Steve Tseng, Lei Ye, John F. Flaherty, Anu Osinusi, Ben L. Da, Grace M. Chee, Audrey H. Lau, Maurizia R. Brunetto, Heiner Wedemeyer

**Affiliations:** ^1^ Department of Hepatology, Hôpital Beaujon, Université de Paris‐Cité, INSERM UMR1149 Clichy France; ^2^ Division of Gastroenterology and Hepatology Foundation IRCCS Ca' Granda Ospedale Maggiore Policlinico Milan Italy; ^3^ CRC ‘A. M. And A. Migliavacca’ Center for Liver Disease, Department of Pathophysiology and Transplantation University of Milan Milan Italy; ^4^ Department of Infectious Diseases Karolinska University Hospital/Karolinska Institutet Stockholm Sweden; ^5^ Hôpital Saint Joseph Marseille France; ^6^ National Institute for Infectious Diseases ‘Prof. Dr. Matei Bals’ Bucharest Romania; ^7^ Carol Davila Medicine and Pharmacy University Bucharest Romania; ^8^ M.F. Vladimirsky Moscow Regional Research and Clinical Institute Moscow Russian Federation; ^9^ LLC Medical Company ‘Hepatolog’ Samara Russian Federation; ^10^ Limited Liability Company ‘Clinic of Modern Medicine’ Moscow Russian Federation; ^11^ Dr. Victor Babes Foundation Bucharest Romania; ^12^ Gilead Sciences Inc. Foster City California USA; ^13^ Hepatology Unit, Reference Center of the Tuscany Region for Chronic Liver Disease and Cancer University Hospital of Pisa Pisa Italy; ^14^ Department of Clinical and Experimental Medicine University of Pisa Pisa Italy; ^15^ Clinic for Gastroenterology, Hepatology, Infectious Diseases, and Endocrinology Hannover Medical School Hannover Germany

**Keywords:** antiviral agent, bulevirtide, hepatitis B virus, hepatitis D virus, Hepcludex, safety

## Abstract

**Background and Aims:**

The safety and tolerability of bulevirtide (BLV), a novel entry inhibitor of hepatitis delta virus, were evaluated in an integrated analysis of clinical trial results from patients with chronic hepatitis delta (CHD).

**Methods:**

Week 48 on‐treatment clinical and laboratory results from two Phase 2 trials (MYR203 [NCT02888106] and MYR204 [NCT03852433]) and one Phase 3 trial (MYR301 [NCT03852719]) were pooled (*N* = 269). Patients were grouped as follows: BLV 2 mg (*n* = 64), BLV 10 mg (*n* = 115), pegylated interferon‐alfa (*n* = 39) and control (*n* = 51). The control group consisted of patients assigned to the delayed treatment group in Study MYR301.

**Results:**

Adverse events (AEs) that occurred more frequently with BLV 2 mg and BLV 10 mg versus control included increased total bile acid levels (20% and 17% vs. 0%), injection‐site reactions (16% and 20% vs. 0%), headache (16% and 17% vs. 0%), pruritus (11% and 10% vs. 0%) and eosinophilia (9% and 4% vs. 0%). Increases in total bile acid levels were observed with BLV without clear correlation with AEs, such as pruritus, eosinophilia or vitamin D deficiency. Grade 3 or 4 study drug–related AEs occurred in a higher proportion of patients receiving pegylated interferon‐alfa (51%) than with BLV 2 or 10 mg (3% and 4%, respectively). There were no serious AEs related to BLV, and no patients discontinued BLV due to an AE. Neither hepatic decompensation nor death occurred.

**Conclusions:**

BLV monotherapy was safe and well tolerated through 48 weeks of treatment in patients with CHD.

**Trial Registration:** NCT02888106, NCT03852433 and NCT03852719

**Summary**

- Hepatitis delta virus infection causes the most severe form of chronic viral hepatitis.
- Bulevirtide has been shown to effectively treat chronic hepatitis delta infection.
- In this integrated analysis of clinical trials, we demonstrate that treatment with bulevirtide for 48 weeks was safe and well tolerated among 218 patients who had chronic hepatitis delta infection.

**Abbreviations**
AE: adverse event
ALT: alanine aminotransferase
AST: aspartate aminotransferase
BL: baseline
BLV: bulevirtide
BMI: body mass index
CHD: chronic hepatitis delta
COVID‐19: coronavirus disease 2019
CTCAE: Common Terminology Criteria for Adverse Events
DILI: drug‐induced liver injury
eDISH: evidence of drug‐induced serious hepatotoxicity
EMA: European Medicines Agency
GGT: gamma‐glutamyl transferase
HBeAg: hepatitis B e antigen
HBsAg: hepatitis B virus surface antigen
HBV: hepatitis B virus
HDV: hepatitis delta virus
HLT: high‐level term
ISR: injection‐site reaction
MedDRA: Medical Dictionary for Regulatory Activities
NA: nucleos(t)ide analogue
NTCP: sodium taurocholate cotransporting polypeptide
Peg‐IFNα: pegylated interferon‐alfa
PT: preferred term
Q1: first quartile
Q3: third quartile
SAE: serious adverse event
SoC: standard of care
TBL: total bilirubin
ULN: upper limit of normal

## 1 Introduction

Hepatitis delta virus (HDV) causes chronic hepatitis delta (CHD), which represents the most severe form of chronic viral hepatitis [1]. HDV is a satellite virus of hepatitis B virus (HBV) requiring its surface antigen (HBsAg) for hepatocyte entry and propagation. The exact prevalence of CHD is uncertain; however, it is estimated to affect approximately 10–20 million people worldwide [2]. Progression from CHD to cirrhosis occurs more rapidly than with HBV infection alone, occurring in approximately 30% of infected individuals within 3 years [3, 4]. Thus, every effort should be made to screen and identify people at risk for CHD as they are at increased risk of liver‐related events, such as hepatic decompensation, hepatocellular carcinoma and liver‐related mortality, compared to chronic infection with HBV alone [5, 6, 7].

Despite the serious nature of HDV infection, effective and tolerable treatment options have been lacking since its discovery almost 5 decades ago [8]. Until recently, the only therapeutic option was pegylated interferon‐alfa (Peg‐IFNα), which is used off‐label for this indication and can be poorly tolerated [9]. Treatment with Peg‐IFNα has been reported to result in rates of virologic response (undetectable HDV RNA at 6 months posttreatment) of 17%–43% [9, 10]. However, these rates may be overestimated due to the lack of sensitive assays at the time and to a high risk of late relapse [11]. For patients with end‐stage liver disease who cannot tolerate Peg‐IFNα or who are nonresponsive to this therapy, the only option to prevent disease progression and death has been liver transplantation [12].

Bulevirtide (BLV), a synthetic lipopeptide that mimics the preS1 domain of HBsAg, effectively works as an entry inhibitor of both HBV and HDV by blocking the entry of these viruses via binding and inactivation of the sodium taurocholate cotransporting polypeptide (NTCP) receptor found on the basolateral membrane of hepatocytes [13].

The EMA fully approved BLV in July 2023 for the treatment of CHD in adult patients with compensated liver disease [14]. Final approval was based on Week 48 results from Study MYR301, a Phase 3 randomised, open‐label trial of BLV monotherapy [15]. Forty‐five per cent and 48% of patients in the BLV 2‐ and 10‐mg treatment groups, respectively, achieved combined virologic (undetectable HDV RNA or a ≥ 2 log_10_ IU/mL HDV RNA decline from baseline) and biochemical (normalisation of serum alanine aminotransferase [ALT] level) response compared to only 2% in the control group with delayed treatment at Week 48. Viral resistance analysis has shown no development of resistance at either dosage of BLV (2 or 10 mg), and real‐world data, including among patients with compensated cirrhosis, have confirmed these findings [16, 17]. Following the approval of BLV in Europe, further evidence has confirmed the efficacy and safety of BLV [18]. As a result, the recently released European Association for the Study of the Liver guidelines on CHD recommend BLV be considered for all patients with CHD and active HDV replication wherever available [19].

In this integrated analysis of data derived from completed and ongoing clinical trials, we describe the safety and tolerability of BLV therapy after 48 weeks of treatment among patients with CHD and compensated liver disease.

## 2 Materials and Methods

### 2.1 Patients

**FIGURE 1:**
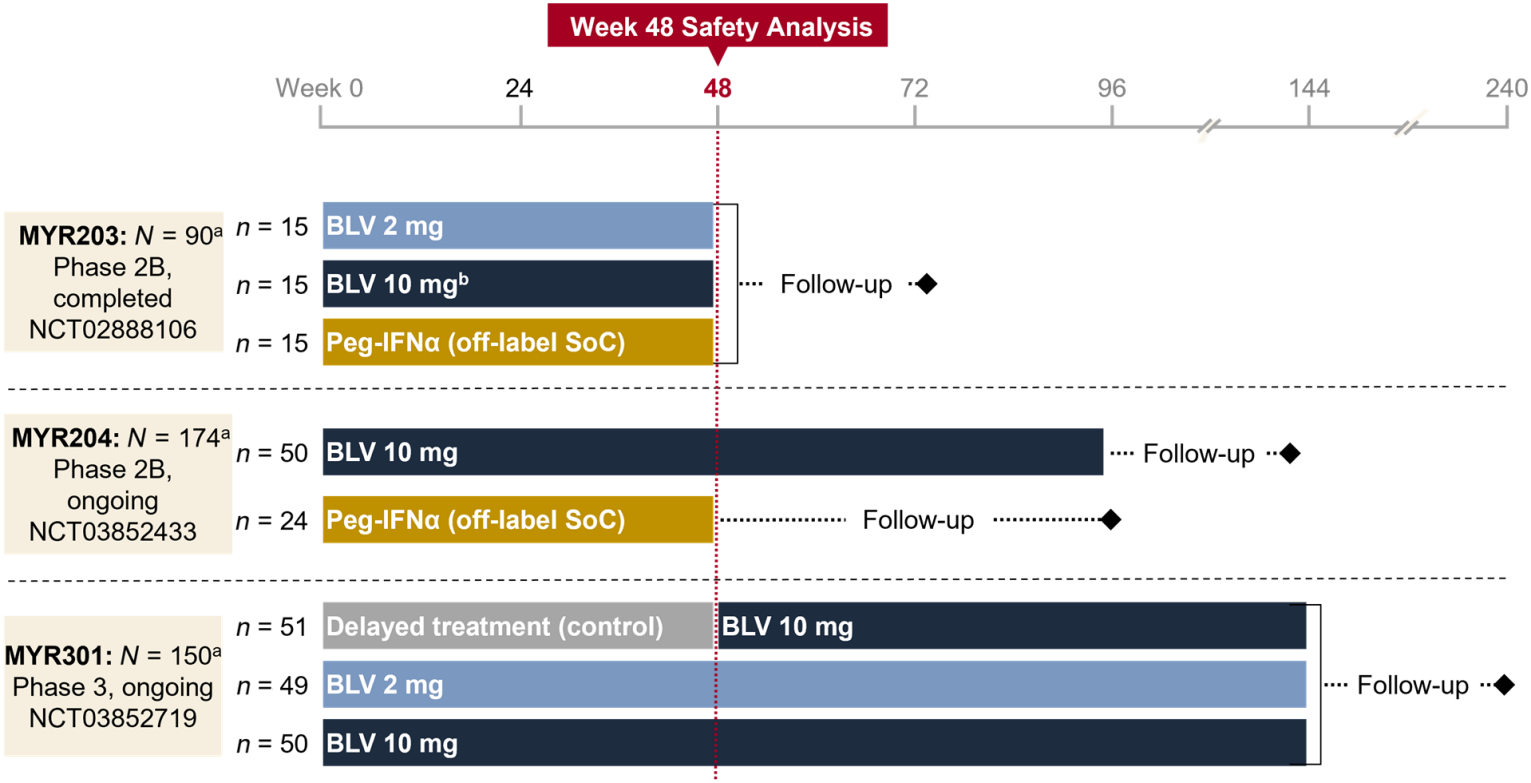
Bulevirtide integrated safety analysis. Study arms pooled for the Week 48 safety analysis set are shown; data from the 24‐week treatment‐free follow‐up period of MYR203 were included for certain analyses of the adverse event of interest and total bile acids elevation. ^a^Total *N* of study patients treated. ^b^In combination with tenofovir disoproxil fumarate. BLV, bulevirtide; Peg‐IFNα, pegylated interferon‐alfa; SoC, standard of care. Primary report of MYR204 published as Asselah et al. [20]; primary report of MYR301 published as Wedemeyer et al. [15].

All available safety data were pooled (Week 48 safety analysis sets) from three multicentre, open‐label, randomised Phase 2 and 3 clinical trials for patients with CHD treated with BLV (2 or 10 mg) or Peg‐IFNα (reference group) as monotherapy for 48 weeks or who received no anti‐HDV treatment (delayed‐treatment group [control] of MYR301; Figure 1; Table S1). The 10‐mg dosage of BLV was administered as two subcutaneous (s.c.) injections of 5 mg each, while the 2‐mg dosage was given as a single injection. Studies MYR203 (NCT02888106), MYR204 (NCT03852433) [20] and MYR301 (NCT03852719) [15] were conducted in seven countries: France, Germany, Italy, Moldova, Romania, Russian Federation and Sweden. All research was conducted in accordance with the Declarations of Helsinki and Istanbul and approved by the appropriate ethics and review committees. Additionally, all patients in these studies provided written, informed consent. Reporting of data is in accordance with CONSORT reporting guidelines [21].

Key inclusion criteria included men and women aged 18–65 years, detectable HDV RNA at screening, CHD with or without compensated cirrhosis and elevated ALT (Table S2). Exclusion criteria included any history of decompensated cirrhosis (MYR203 and MYR204) or decompensated cirrhosis within 2 years (MYR301), platelet count < 90 000 cells/mm^3^ (MYR203 and MYR204) or < 60 000 cells/mm^3^ (MYR301), active coinfection with hepatitis C virus, creatinine clearance < 60 mL/min and total serum bilirubin ≥ 34.2 μmol/L. Nucleos(t)ide analogue (NA) therapy was permitted in Studies MYR204 and MYR301 if indicated according to HBV treatment guidelines [22, 23]. Notably, NAs were not used in MYR203, except in the BLV 10‐mg monotherapy treatment arm (*n* = 15), for which concomitant use of tenofovir disoproxil fumarate was included in the study design.

For the completed Study MYR203, all safety data are included in the analysis. For the two ongoing Studies MYR204 and MYR301, all safety data up to the Week 48 visit for each patient are included in the analysis. Unless otherwise specified, the same methods used for the analyses of safety specified in each individual study statistical analysis plan and/or protocol are applied to the analysis presented here. All data are summarised by treatment group and overall using descriptive statistics. As in the individual studies, no formal statistical comparisons between treatment groups were performed.

### 2.2 Assessments

#### 2.2.1 Increases in Total Bile Acids

Increases in serum total bile acids are expected with BLV treatment due to its mechanism of action, which is the inhibition of the bile acid transporter NTCP. Blood samples were collected to assess serum total bile acid levels at every study visit. Reporting of increased total bile acids as an adverse event (AE) on treatment differed by study. In the early Phase 2 study MYR203, the protocols did not contain specific instructions for investigators on this matter. In contrast, for Studies MYR204 and MYR301, investigators were instructed to report only symptomatic or otherwise clinically meaningful elevations in total bile acids as AEs.

#### 2.2.2 Assessment of Injection‐Site Reactions

In the BLV monotherapy groups, injection‐site reactions (ISRs) were reported by the grouped term ‘injection‐site reactions’, which included the following preferred terms per Medical Dictionary for Regulatory Activities (version 24.0): ‘Injection site’ plus any one of ‘coldness’, ‘dermatitis’, ‘discoloration’, ‘erythema’, ‘extravasation’, ‘hematoma’, ‘induration’, ‘irritation’, ‘pain’, ‘pruritus’, ‘rash’, ‘reaction’, or ‘swelling’.

#### 2.2.3 Assessment of Potential for Drug‐Induced Liver Injury

An analysis for the possibility of drug‐induced liver injury (DILI) with BLV was performed using a set of laboratory‐based criteria derived from Hy's law [24] during the on‐treatment period. Patients who met any one of these criteria were evaluated for DILI.

- Criterion 1: ALT and/or aspartate aminotransferase (AST) > 3 × upper limit of normal (ULN) and total bilirubin > 2 × ULN
- Criterion 2: ALT > 5 × ULN
- Criterion 3: Total bilirubin > 2 × ULN

## 3 Results

### 3.1 Patient Population

Patient disposition for those included in the integrated safety analysis set is shown in Table S3, and demographic and baseline clinical characteristics are provided in Table 1. A total of 269 patients were included in the main safety analysis set consisting of 218 who received active treatment (BLV 2 mg, *n* = 64; BLV 10 mg, *n* = 115; and Peg‐IFNα, *n* = 39) and 51 untreated patients in the control group. Baseline demographics were fairly well balanced across treatment groups. Cirrhosis was present in approximately one‐third to one‐half of patients and was lower in studies that included Peg‐IFNα. Nearly all patients were HDV genotype 1.

**TABLE 1:** Patient demographics and baseline characteristics of the safety analysis set.

	Control (*n* = 51)	BLV 2 mg (*n* = 64)	BLV 10 mg (*n* = 115)	Peg‐IFNα (*n* = 39)
Age (years), mean (range)	41 (27–61)	43 (19–62)	40 (18–62)	38 (20–59)
Male, *n* (%)	26 (51)	41 (64)	79 (69)	23 (59)
BMI (kg/m^2^), mean (SD)	25.3 (3.9)	25.0 (3.5)	25.1 (3.9)	25.5 (4.2)
BMI ≥ 30 kg/m^2^, *n* (%)	5 (10)	5 (8)	12 (10)	5 (13)
Race, *n* (%)				
White	40 (78)	56 (88)	102 (89)	34 (87)
Asian	11 (22)	8 (13)	10 (9)	5 (13)
Black	0	0	3 (3)	0
Other	0	0	0	0
Cirrhosis present, *n* (%)	24 (47)	26 (41)	41 (36)	12 (31)
Liver stiffness (kPa), mean (SD)	15 (9.0)	14 (7.8)	13 (7.8)	14 (9.8)
HDV RNA (log_10_ IU/mL), mean (SD)	5.1 (1.4)	5.2 (1.3)	5.3 (1.4)	5.1 (1.1)
HDV genotype, *n* (%)				
1	51 (100)	64 (100)	109 (95)^b^	38 (97)^b^
5	0 (0)	0 (0)	2 (2)	0 (0)
HBeAg negative, *n* (%)	47 (92)	57 (89)	101 (88)	37 (95)
HBsAg (log_10_ U/mL), mean (SD)	3.7 (0.5)	3.8 (0.5)	3.7 (0.7)	3.8 (0.5)
HBV DNA (log_10_ IU/mL), mean (SD)	0.9 (1.0)	1.4 (1.3)	1.4 (1.5)	1.3 (1.6)
Patients with positive HBV DNA, *n* (%)	27 (53)	44 (69)	80 (70)	24 (62)
HBV DNA (log_10_ IU/mL), among patients with positive HBV, mean (SD)	1.7 (0.7)	2.0 (1.2)	2.0 (1.4)	2.1 (1.5)
ALT (U/L), mean (SD)	102 (62)	111 (73)	115 (90)	110 (80)
Creatinine clearance category, *n* (%)				
≥ 60–< 90 mL/min	10 (20)	10 (16)	14 (12)	4 (10)
≥ 90 mL/min	41 (80)	54 (84)	101 (88)	35 (90)
Vitamin D level (ng/mL), mean (SD)^a^	25.8 (12.22)	28.1 (12.28)^b^	27.8 (13.35)^b^	25.9 (10.19)
Total bile acids (μmol/L), mean (SD)	15.8 (11.94)	14.0 (13.27)	13.4 (10.78)	10.8 (8.84)
Nucleos(t)ide use, *n* (%)	32 (63)	31 (48)	65 (57)	11 (28)
Previous interferon therapy, *n* (%)	29 (57)	27 (42)	50 (44)	16 (41)

*Note:* Data from MYR203, MYR204 and MYR301 are included.

Abbreviations: ALT, alanine aminotransferase; BLV, bulevirtide; BMI, body mass index; HBeAg, hepatitis B e antigen; HBsAg, hepatitis B surface antigen; HBV, hepatitis B virus; HDV, hepatitis delta virus; Peg‐IFNα, pegylated interferon‐alfa.

^a^ Data missing or negative for < 5% of patients.

^b^ Vitamin D level was assessed only in Studies MYR204 and MYR301.

### 3.2 Overall AEs

Treatment with BLV for 48 weeks was generally safe and well tolerated. Table 2 summarises the AEs by treatment group. The following AEs occurred more often among patients who received BLV 2 mg (*n* = 64)—the approved dosage for treatment of CHD in adults with compensated liver disease [14]—compared to controls: total bile acids increased (13 patients, 20%), ISRs (10 patients, 16%), headache (10 patients, 16%), pruritus (7 patients, 11%), eosinophilia (6 patients, 9%), neutropenia (5 patients, 8%), ALT increased, (5 patients, 8%) and fatigue (6 patients, 9%).

**TABLE 2:** AEs, discontinuations and laboratory abnormalities by Week 48.

	Control (*n* = 51), *n* (%)	BLV 2 mg (*n* = 64), *n* (%)	BLV 10 mg (*n* = 115), *n* (%)	Peg‐IFNα (*n* = 39), *n* (%)
Any AE	39 (77)	55 (86)	99 (86)	35 (90)
Grade ≥ 3 AE	3 (6)	7 (11)	13 (11)	20 (51)
Serious AE	1 (2)	2 (3)	2 (2)	3 (8)
Related to study drug	—	38 (59)	72 (63)	34 (87)
AE related to study drug Grade ≥ 3	—	2 (3)	5 (4)	20 (51)
Discontinuations of study drug due to AE	—	0	0	3 (8)
Death	0	0	0	0
Common AEs^a^				
Total bile acids increased	0	13 (20)^b^	19 (17)^b^	6 (15)^b^
Injection‐site reaction^c^	0	10 (16)	23 (20)	1 (3)
Headache	0	10 (16)	19 (17)	5 (13)
Leukopenia	9 (18)	10 (16)	16 (14)	22 (56)
Thrombocytopenia	8 (16)	8 (13)	15 (13)	22 (56)
Pruritus	0	7 (11)	11 (10)	2 (5)
Eosinophilia	0	6 (9)	5 (4)	1 (3)
Vitamin D deficiency	8 (16)	6 (9)	18 (16)	3 (8)
Neutropenia	3 (6)	5 (8)	14 (12)	20 (51)
ALT increased	3 (6)	5 (8)	9 (8)	14 (36)
Lymphopenia	4 (8)	4 (6)	9 (8)	12 (31)
Nausea	2 (4)	3 (5)	9 (8)	6 (15)
Fatigue	1 (2)	6 (9)	8 (7)	2 (5)
Laboratory abnormality	42 (82)	58 (91)	100 (87)	37 (100)
Grade ≥ 3	6 (12)	13 (20)	16 (14)	25 (68)

*Note:* Multiple AEs were counted only once per patient for the highest severity grade for each PT. Except for the ‘injection‐site reaction’ grouped term, PTs were presented by descending order of the total frequencies.

Abbreviations: AE, adverse event; ALT, alanine aminotransferase; BLV, bulevirtide; HLT, high‐level term; MedDRA, Medical Dictionary for Regulatory Activities; Peg‐IFNα, pegylated interferon‐alfa; PT, preferred term.

^a^ Events that occurred in ≥ 7.5% of patients within the BLV 2‐mg or BLV 10‐mg groups.

^b^ In Studies MYR204 and MYR301, only symptomatic or clinically significant (as judged by the investigator) increases in total bile acids were reported as AEs (PT total bile acids increased) per protocol; there were no Grade 3 or higher AEs of total bile acids increased reported in those studies.

^c^ Grouped term includes any PT under the MedDRA HLT injection‐site reactions.

In general, the incidences of BLV‐related AEs were similar between the BLV 2‐ and 10‐mg groups (BLV 2 mg, 59% vs. BLV 10 mg, 63%), except for ISRs, which occurred in a slightly higher proportion of patients treated with BLV 10 mg (20%) compared to those treated with BLV 2 mg (16%). This is likely due to the need for two 5‐mg injections of BLV daily to deliver the higher dosage. A summary of ISRs is included below under AEs of special interest. Most AEs seen with BLV 2 or 10 mg were Grade 1 or 2. No patients discontinued BLV 2 or 10 mg due to an AE; this is in contrast to the Peg‐IFNα monotherapy group, from which 3 of 39 (8%) patients discontinued therapy due to an AE. Comparatively, a higher proportion of drug‐related AEs was seen among patients treated with Peg‐IFNα monotherapy (87%).

### 3.3 Grade 3 or 4 AEs and Serious AEs

Grade 3 or higher AEs were experienced by 3 of 51 (6%) patients in the control group compared to 7 of 64 (11%) treated with BLV 2 mg and 13 of 115 (11%) treated with BLV 10 mg (Table 3). Fifty‐one per cent of patients (20 of 39) treated with Peg‐IFNα monotherapy experienced a Grade 3 or higher AE. A Grade 3 or higher AE that was considered related to BLV occurred in two patients (3%) treated with BLV 2 mg and five patients (4%) treated with BLV 10 mg. In the BLV 2‐mg group, these events were Grade 3 AST increased (*n* = 1) and neutrophil count decreased (*n* = 1); in the BLV 10‐mg group, these events were Grade 3 neutropenia (*n* = 1), leukopenia (*n* = 1), thrombocytopenia (*n* = 1) and total bile acids increased (*n* = 2).

**TABLE 3:** Grade 3 or 4 AEs and SAEs by Week 48.

	Control (*n* = 51), *n* (%)	BLV 2 mg (*n* = 64), *n* (%)	BLV 10 mg (*n* = 115), *n* (%)	Peg‐IFNα (*n* = 39), *n* (%)
Grade ≥ 3 AE	3 (6)	7 (11)	13 (11)	20 (51)
Neutropenia	2 (4)	0	4 (3)	10 (26)
Leukopenia	1 (2)	0	2 (2)	6 (15)
Thrombocytopenia	2 (4)	1 (2)	3 (3)	5 (13)
Neutrophil count decreased	0	1 (2)	0	2 (5)
ALT increased	0	1 (2)	0	4 (10)
AST increased	0	1 (2)	0	3 (8)
GGT increased	0	0	0	3 (8)
Total bile acids increased	0	0^a^	2 (2)^a^	1 (3)^a^
Serious AEs	1 (2)	2 (3)	2 (2)	3 (8)
Pyrexia	0	0	0	1 (3)
Cholelithiasis	1 (2)	0	0	0
Asthenia	0	1 (2)	0	0
Appendicitis	0	0	0	1 (3)
Urinary tract infection	0	0	1 (1)	0
COVID‐19	1 (2)	0	1 (1)	1 (3)
Depression	0	1 (2)	0	0
Foot fracture	0	1 (2)	0	0

*Note:* AEs were coded according to MedDRA Version 24.0. Multiple AEs were counted only once per patient for the highest severity grade for each PT. Severity grades were defined by the CTCAE. The control group corresponds to the MYR301 delayed‐treatment group. In Studies MYR204 and MYR301, only symptomatic or clinically significant (as judged by the investigator) increases in total bile acids were reported as AEs (PT total bile acids increased) per protocol; there were no Grade 3 or higher AEs of total bile acids increased reported in those studies.

Abbreviations: AE, adverse event; ALT, alanine aminotransferase; AST, aspartate aminotransferase; BLV, bulevirtide; COVID‐19, coronavirus disease 2019; CTCAE, Common Terminology Criteria for Adverse Events; GGT, gamma‐glutamyl transferase; MedDRA, Medical Dictionary for Regulatory Activities; Peg‐IFNα, pegylated interferon‐alfa; PT, preferred term; SAE, serious adverse event.

^a^ All patients who experienced Grade 3 or higher AEs of total bile acids increased were from Study MYR203.

The incidences of serious AEs (SAEs) were similar between the control group (*n* = 1, 2%) and the BLV treatment groups (2 mg: *n* = 2, 3%; 10 mg: *n* = 2, 2%) compared with patients in the Peg‐IFNα group (*n* = 3, 8%). None of the SAEs that occurred in the BLV treatment groups were considered related to BLV; there were no deaths in the analysis population.

### 3.4 AEs of Special Interest

#### 3.4.1 Bile Acid Elevations

A similar incidence of the AE of total bile acids increased was reported with BLV 2 and 10 mg (20%, 13 of 64 patients, vs. 17%, 19 of 115; Table 2). Two such AEs were Grade 3 (both in patients treated with BLV 10 mg), and all were asymptomatic. There was no discontinuation of BLV due to the AE of total bile acids increased. Posttreatment follow‐up from MYR203 revealed that all AEs that occurred on treatment had resolved.

**FIGURE 2:**
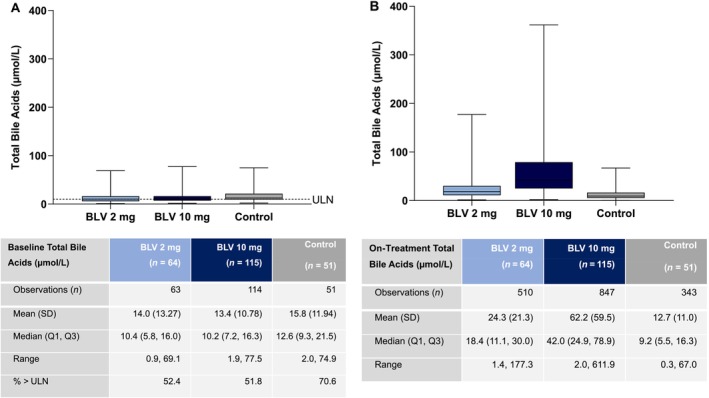
Total bile acid levels at baseline and on treatment. (A) Box plot of baseline total bile acids (μmol/L) from patients who underwent treatment with BLV 2 and 10 mg or delayed treatment (control). The baseline total bile acid value is the last available value collected prior to the first dose of the study drug. (B) Box plot of total bile acids (μmol/L) from on‐treatment visits for the BLV 2‐mg and 10‐mg treatment groups and the delayed‐treatment group (control). For both box plots, the horizontal lines within the shaded boxes represent the medians. The shaded boxes represent the interquartile ranges (25%–75%). The box plot whiskers represent the maximums and minimums. An outlier bile acids value for one participant in the BLV 10‐mg group at Week 8 with a value of 611.9 μmol/L was excluded from the box plot. All on‐treatment visits in Studies MYR203, MYR204 and MYR301 up to Week 48 were included. BLV, bulevirtide; Q1, first quartile; Q3, third quartile; ULN, upper limit of normal.

**FIGURE 3:**
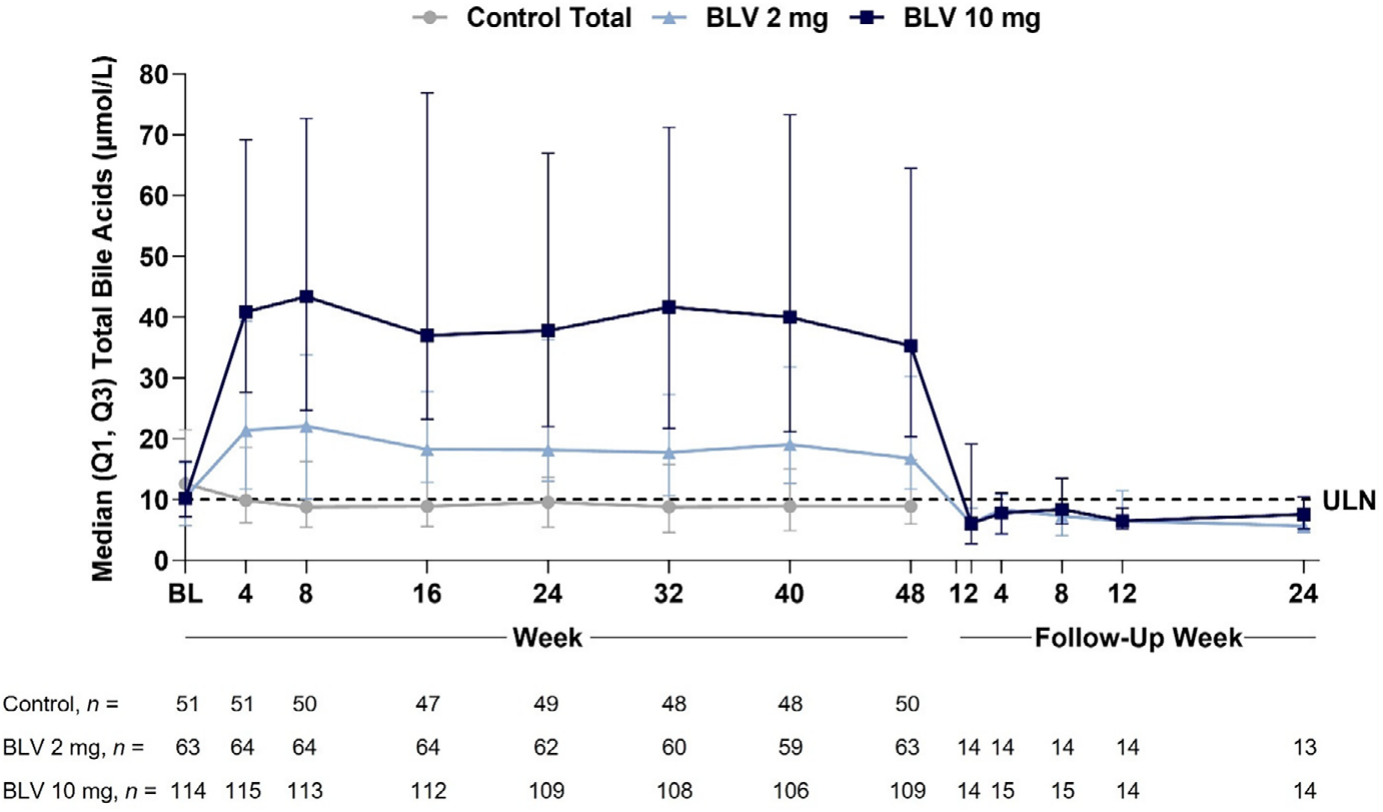
Total bile acids (μmol/L) by visit and treatment group. Data expressed as median (Q1, Q3). This analysis included on‐treatment data from MYR203, MYR204 and MYR301 and follow‐up data from MYR203. The dotted horizontal line represents the ULN. The baseline value was the last available value collected prior to the first dose of the study drug. For patients in the Study MYR301 delayed‐treatment group, the baseline value was the last available value collected at or prior to randomisation. The control group in this figure corresponds to the MYR301 delayed‐treatment group. BL, baseline; BLV, bulevirtide; Q1, first quartile; Q3, third quartile; ULN, upper limit of normal.

Approximately one‐half of patients in the BLV 2‐ and 10‐mg groups had total serum bile acid levels > ULN at baseline (52% in each group; 71% in controls; Figure 2A,B). With BLV treatment, total bile acids increased rapidly in a dose‐dependent fashion and plateaued quickly after treatment initiation with considerable intragroup variability (Figure 3). The median (quartile [Q]1 and Q3) changes in total bile acids from baseline to Week 48 for BLV 2 mg compared to 10 mg were +6.6 (0.4, 21.2) μmol/L versus +24.1 (7.2, 52.5) μmol/L. At Week 48, 79% and 94% of patients treated with BLV 2 and 10 mg had total bile acid levels > ULN, respectively. In comparison, 71% and 37% of patients in the control and Peg‐IFNα monotherapy groups had total bile acid levels > ULN at baseline, and an increase in total bile acid levels from baseline was not observed in the control and Peg‐IFNα monotherapy treatment groups. Posttreatment data from study MYR203 showed that total bile acids returned to baseline levels by the first follow‐up visit, 2 weeks following BLV discontinuation (Figure 3).

**FIGURE 4:**
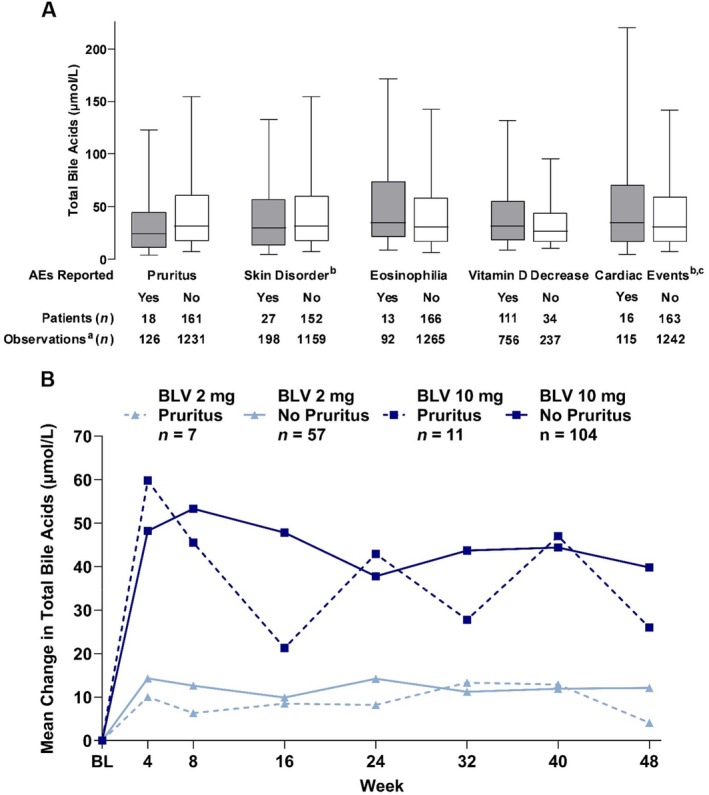
Relationship of total bile acid levels with AEs of interest. For the box plots, the horizontal lines within the shaded boxes represent the medians. The shaded boxes represent the interquartile ranges (25%–75%). The box plot whiskers represent the 5th and 95th percentiles. (A) Box plots of the total bile acid levels in patients with and without AEs of interest (BLV 2 and 10 mg combined). (B) Mean change in total bile acid levels over time in patients with and without pruritus. ^a^N represents individual records of on‐treatment bile acid level values. ^b^Identified by system organ class term according to MedDRA v. 24.0. ^c^Most common cardiac events were bradycardia (*n* = 8) and tachycardia (*n* = 3), all asymptomatic, Grade 1–2. AE, adverse event; BL, baseline; BLV, bulevirtide; MedDRA, Medical Dictionary for Regulatory Activities.

There was no clear relationship between increased total bile acid levels during BLV treatment and any symptoms or clinical sequelae, including specific AEs of interest, such as pruritus, skin disorders, eosinophilia, vitamin D decrease and cardiac events (Figure 4A,B). Changes in total bile acid levels were numerically similar between patients with and without pruritus (Table S4). Among patients who received BLV 2 mg, there were 9 AEs of pruritus, only one of which occurred concurrently with peak total bile acid levels; likewise, of 17 AEs of pruritus with BLV 10 mg, 3 occurred concurrently with peak total bile acid levels. Thus, although pruritus was more common among those treated with BLV 2 or 10 mg than among controls or those treated with Peg‐IFNα, there was no clear association between total bile acid levels and pruritus onset, duration or severity (Figure S1). Total bile acid levels also did not correlate with ALT (Figure S2), and no major difference in total bile acid levels was observed between patients with and without cirrhosis (Figure S3). Elevated total bile acids with 48‐week BLV treatment can be considered asymptomatic, unrelated to clinical sequelae and rapidly reversible after treatment cessation.

#### 3.4.2 Injection‐Site Reactions

A slightly higher rate of ISRs was seen with BLV 10 mg than with BLV 2 mg (20%, 23 of 115, vs. 16%, 10 of 64; Table 2). The median (Q1 and Q3) duration of the ISRs was longer with BLV 10 mg (15 [1, 59] days) compared with BLV 2 mg (9 [2, 42] days). All ISRs were Grade 1 or 2, none were considered an SAE and none led to BLV discontinuation.

#### 3.4.3 Eosinophilia

Eosinophilia occurred in 6 of 64 (9%) and 5 of 115 (4%) patients treated with BLV 2 and 10 mg. In all 11 patients, the eosinophilia was mild in severity and resolved while still on treatment. No patients with eosinophilia met the criteria for potential DILI.

#### 3.4.4 Vitamin D Deficiency

Vitamin D levels were assessed only in the MYR204 and MYR301 studies. Mean vitamin D levels were similar across treatment groups at baseline (Table 1). At Week 48, the AE of vitamin D deficiency occurred less frequently among those treated with BLV 2 mg (6 patients, 9%) compared to controls (8 patients, 16%) or those treated with BLV 10 mg (18 patients, 16%). However, the mean (SD) change in vitamin D levels across the control (+0.9 [10.2] ng/mL), BLV 2‐mg (−0.2 [10.2] ng/mL) and BLV 10‐mg (+1.8 [10.2] ng/mL) groups at Week 48 were similar and minimal (Table S5). In contrast, vitamin D levels increased in the Peg‐IFNα monotherapy group at Week 48 (Table S5). Taken together, the incidence of vitamin D deficiency and the absolute levels of vitamin D were similar among patients with CHD treated with BLV and those without BLV treatment.

#### 3.4.5 On‐Treatment ALT Elevations and Assessment of Potential for DILI

**FIGURE 5:**
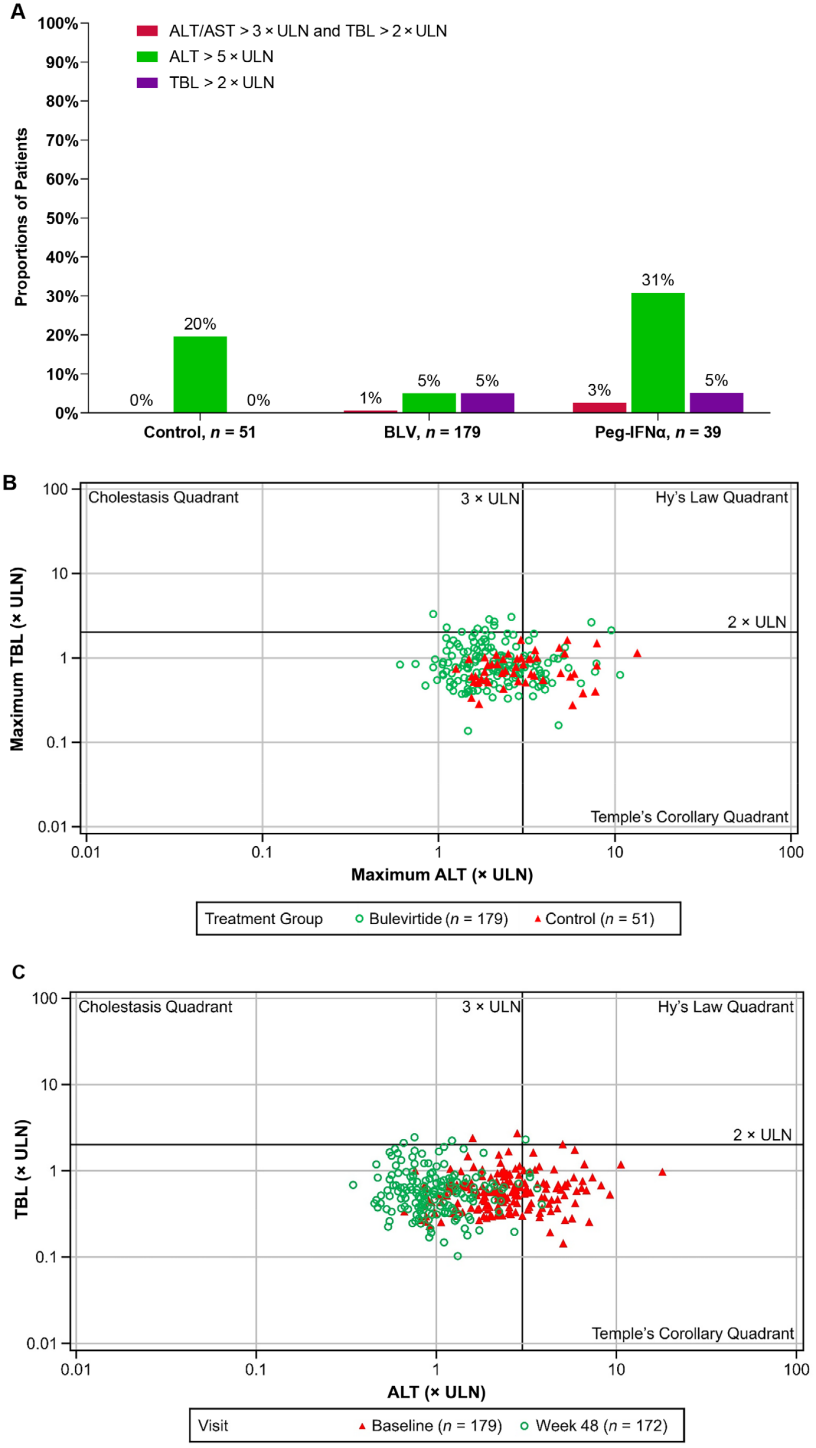
Liver test abnormalities while on treatment. On‐treatment data from MYR203, MYR204 and MYR301 are included in (A) and (B). Baseline and on‐treatment data from MYR203, MYR204 and MYR301 are included in Panel (C). (A) Lower incidence of liver test abnormalities with BLV. (B) eDISH plot of maximum TBL and ALT levels. (C) eDISH plot of baseline compared to Week 48 TBL and ALT levels. The control group in (A) and (B) corresponds to the MYR301 delayed‐treatment group. The BLV group in (A) and (B) included patients who received BLV 2 or 10 mg as monotherapy. The BL value was the last available value collected prior to the first dose of the study drug. For patients in the study MYR301 delayed‐treatment group, the BL value was the last available value collected at or prior to randomisation. ALT, alanine aminotransferase; AST, aspartate aminotransferase; BL, baseline; BLV, bulevirtide; eDISH, evidence of drug‐induced serious hepatotoxicity; Peg‐IFNα, pegylated interferon‐alfa; TBL, total bilirubin; ULN, upper limit of normal.

BLV treatment at either dosage level was not associated with on‐treatment ALT elevations or DILI. Figure 5A depicts the patterns and incidence of liver test abnormalities between the treatment groups. This figure shows a lower incidence of liver test abnormalities overall among those treated with BLV compared to controls.

Similar proportions of patients in the BLV 2‐ and 10‐mg groups met at least one criterion for potential DILI on treatment (BLV 2 mg, 12.5%; BLV 10 mg, 7.0%). One patient in the BLV 10‐mg monotherapy group met Criterion 1 for potential DILI (ALT and/or AST > 3 × ULN and total bilirubin > 2 × ULN; Figure 5B). After evaluation by an independent hepatic safety adjudication committee, the liver test abnormalities on BLV treatment that led to a positive criterion for potential DILI were attributed to the underlying liver disease or relevant medical history, and no patient met Hy's law criteria. Hepatic decompensation also did not occur in any patient. Baseline liver test abnormalities improved with treatment at Week 48 (Figure 5C).

## 4 Discussion

BLV, a novel entry inhibitor, has demonstrated efficacy and safety in patients with CHD, including results from a Phase 3 randomised, open‐label trial. In this integrated Week 48 analysis, the safety results of BLV monotherapy from multiple studies were combined to obtain a more comprehensive review of its safety profile, including comparisons to a delayed‐treatment group that served as the control and a reference group receiving Peg‐IFNα monotherapy. BLV monotherapy at both dosages (2 and 10 mg/day) was safe and well tolerated. The vast majority (> 95%) of AEs related to BLV treatment were mild or moderate (i.e., Grade 1 or 2) in severity with no SAEs that were related to BLV and no discontinuations of BLV due to an AE. The favourable safety profile of BLV has been confirmed in multiple real‐world studies from countries in which BLV has been available [18, 25, 26]. Moreover, both dosages of BLV monotherapy (2 or 10 mg/day) demonstrated a favourable safety/tolerability profile compared to Peg‐IFNα.

As a synthetic lipopeptide, BLV must be administered subcutaneously, and its elimination half‐life supports once‐daily dosing. Safety and tolerability were found to be generally similar when BLV was given in a daily dosage of 2 or 10 mg, except for ISRs, which occurred more commonly and lasted longer in patients who received BLV 10 mg. This is likely due to the two injections (one more than the 2‐mg dosage) that are required to deliver the 10‐mg dosage; of note, patients were instructed to rotate injection sites in these studies. Importantly, all ISRs were Grade 1 or 2 in severity, and none required discontinuation of BLV, therefore supporting treatment tolerability as confirmed by real‐world experience [27, 28].

The BLV 10‐mg dosage increased total bile acid levels more than the BLV 2‐mg dosage. Increases in serum total bile acids were anticipated based on the known mechanism of action of BLV. In addition to being the central means for HBV and HDV viral entry, the NTCP receptor is the main hepatic uptake transporter for conjugated bile acids as the last step of the enterohepatic bile acid circulation [13]. The results presented here show that the effect of BLV on bile acids is asymptomatic, dose dependent and not associated with any clinical sequelae, including pruritus, eosinophilia and vitamin D deficiency. However, the impact of long‐term elevations in total bile acids is still unknown and requires further study. Studies MYR204 and MYR301 include treatment with BLV as monotherapy for up to 96 and 144 weeks, respectively. Elevated total bile acid levels are common in patients with liver diseases, and secondary transporters naturally exist that can sustain enterohepatic circulation without NTCP function [29, 30]. Although NTCP inhibition with BLV results in elevated serum total bile acids, in preclinical studies, BLV does not result in increased hepatic intracellular bile acid concentrations that are observed with cholestatic liver diseases [31, 32]. This may explain why the pruritus that is associated with BLV does not correlate with the degree or timing of the total bile acid elevations. However, the aetiology of pruritus remains unknown [33]. Since BLV has become commercially available, elevations in total bile acids have not been associated with any clinically concerning signals in postmarketing studies, and a correlation between elevated total bile acid levels and symptoms, such as diarrhoea or pruritus, has not been demonstrated [16]. Consistent with its reversible binding to NTCP, total bile acid levels rapidly decrease to near baseline levels within 1–2 weeks after BLV treatment discontinuation.

Vitamin D deficiency is commonly found among populations with liver disease, especially those with liver cirrhosis. Despite prior reports suggesting an association of vitamin D deficiency with NTCP dysfunction, we did not find evidence of a detrimental effect on vitamin D levels with BLV treatment at either dosage [34, 35].

Finally, we performed in‐depth analyses of clinical and laboratory data to evaluate whether BLV is associated with any risk of on‐treatment liver injury and found that BLV treatment was not associated with a risk of DILI. Rather, BLV clearly improved pre‐existing elevations in liver tests and prevented higher elevations, signifying improvement of HDV‐related necroinflammatory activity in the liver.

The main limitation of this analysis is that the safety data with BLV monotherapy span only 48 weeks of treatment. Longer‐term results from Studies MYR204 and MYR301 will provide further evidence to support the safety profile of BLV. Follow‐up data from these two studies will also inform on the occurrences of posttreatment hepatitis flares, which are not reported in this study, as this analysis focused on the on‐treatment safety of BLV. Another limitation is that we remain bound by the constraints of the individual study designs and their populations. For example, reporting criteria for the AE of bile acids increased varied among studies, which may have influenced interpretation of clinically meaningful elevations. Since BLV studies enrolled mostly White populations with HDV genotype 1 and with compensated liver disease, it is not certain that similar safety results will be observed in other HDV populations, including those with hepatic decompensation and more severe degrees of renal impairment.

In conclusion, this integrated analysis of BLV treatment through 48 weeks did not identify any new safety concerns associated with BLV monotherapy at either dosage relative to previously published data. BLV has been shown to demonstrate a consistent and predictable tolerability and safety profile, which is more favourable than Peg‐IFNα alone.

## Author Contributions

All authors contributed to the collection of data, the interpretation of data and the drafting or revising of the manuscript. Gilead Sciences Inc., the sponsor, had access to all the data, conducted the data analysis and can vouch for the integrity of the data analysis. All authors approved the final version of the manuscript.

## Ethics Statement

All research was conducted in accordance with the Declarations of Helsinki and Istanbul and approved by the appropriate ethics and review committees. Additionally, all patients in these studies provided written, informed consent.

## Conflicts of Interest

T.A. reports being a speaker and investigator for AbbVie; Eiger Biopharmaceuticals; Gilead Sciences Inc.; Janssen; Merck; MYR Pharmaceuticals; and Roche. P.L. reports being an advisor and serving on speaker bureaus for AbbVie; Aligos Therapeutics; Altona Diagnostics; Antios Therapeutics; Eiger Biopharmaceuticals; Gilead Sciences Inc.; Grifols; GSK; Janssen; MYR Pharmaceuticals; Roche Pharma/Diagnostics; Roboscreen GmbH; and Vir Biotechnology. S.A. reports speaking honoraria from AbbVie; Biogen; Gilead Sciences Inc.; and Merck Sharp & Dohme, and research grants from AbbVie and Gilead Sciences Inc. M.B. reports being a speaker and investigator for AbbVie; Eiger Biopharmaceuticals; Gilead Sciences Inc.; Janssen; Merck; MYR Pharmaceuticals; and Roche. A.S.‐C., P.B., V.M., T.S. and S.L. have nothing to disclose. D.M., R.‐C.M., S.T., L.Y., J.F.F., A.O., B.L.D., G.M.C. and A.H.L. are employees of Gilead Sciences Inc. and may hold stock interest in the company. M.R.B. reports being a consultant and serving on speaker bureaus for AbbVie; Eisai‐Merck Sharp & Dohme; Gilead Sciences Inc.; Janssen; and Roche. H.W. reports being a consultant for Abbott; AbbVie; Aligos Therapeutics; Arbutus Biopharma; Boehringer Ingelheim; Bristol Myers Squibb; Dicerna; Gilead Sciences Inc.; Johnson & Johnson/Janssen‐Cilag; Merck/Schering‐Plough; MYR GmbH; Novartis; Roche; Siemens; Transgene; ViiV Healthcare; and Vir Biotechnology, and speaking honoraria from Abbott; AbbVie; Boehringer Ingelheim; Bristol Myers Squibb; Gilead Sciences Inc.; Johnson & Johnson/Janssen‐Cilag; Merck/Schering‐Plough; MYR GmbH; Novartis; Roche; Siemens; Transgene; and ViiV Healthcare.

## Supporting information


Data S1.

## Data Availability

Gilead Sciences Inc. shares anonymised individual patient data on request or as required by law or regulation with qualified external researchers based on submitted curriculum vitae and reflecting no conflict of interest. The request proposal must also include a statistician. Approval of such requests is at the discretion of Gilead Sciences Inc. and is dependent on the nature of the request, the merit of the research proposed, the availability of the data and the intended use of the data. Data requests should be sent to datarequest@gilead.com. Protocols are available via the online supplement.
