# Supplementary material for: Bulevirtide Monotherapy Is Safe and Well Tolerated in Chronic Hepatitis Delta: An Integrated Safety Analysis of Bulevirtide Clinical Trials at Week 48
Source: Liver Int. 2024 Dec 8;45(4):e16174. doi: 10.1111/liv.16174 (PMC11907224; doi:10.1111/liv.16174)
Supplement: Supplementary file 1 — Data S1. [file LIV-45-0-s001.docx]

**Supplementary Material**

**Supplementary Methods**

*Assessments*

Safety

Per protocol, vital signs, concomitant medications, and samples for clinical laboratory testing were taken at the screening, baseline, and treatment visits. Adverse event (AE) reporting was done at all visits. The Medical Dictionary for Regulatory Activities (MedDRA), version 24.0, was used to code for all AEs. AEs and serious AEs (SAEs) were documented in electronic case-report forms and reported in writing on a prespecified SAE form. The investigators followed up with all AEs to report the outcomes until resolution or stabilization. They evaluated AEs and SAEs for seriousness, relatedness, and severity according to the Common Terminology Criteria for Adverse Events (CTCAE) versions 4 and 5. An external hepatic safety adjudication committee (HSAC) independently reviewed all severe AEs and SAEs related to the hepatobiliary system and other significant safety integrated safety analyses, including potential cases of DILI and eosinophilia. This safety analysis was among the control, BLV, and Peg-IFNα monotherapy groups.

### *Study definitions*

All AEs discussed in this study were treatment-emergent and are referred to as AEs unless otherwise specified. Treatment-emergent AEs were defined as: 1) any AEs with an onset date on or after the study drug start date and no later than 30 days after permanent discontinuation of the study drug and 2) any AEs leading to premature discontinuation of the study drug. For the delayed-treatment group (control) in the study MYR301, treatment-emergent AEs were defined as 1) any AEs with an onset date on or after the randomization date and no later than the patient’s week 48 visit date or 2) any AEs with an onset date on or after the randomization date and no later than 30 days after discontinuation date if patients discontinued before the week 48 visit.

**Table S1.** Summary of clinical studies included in the safety analysis.

| **Study,**  **Phase** | **Countries that enrolled patients** | **Duration** | **Treatment group** | **Number of patients treated^a^** |
| --- | --- | --- | --- | --- |

**Completed clinical studies**

| MYR203,  phase 2 | Russia | 48 weeks (follow-up 24 weeks) | **Group A:** Peg-IFNα 180 μg s.c. once weekly for 48 weeks | A, 15 |
| --- | --- | --- | --- | --- |
|  |  |  | **Group В:** BLV 2 mg s.c. once daily + Peg-IFNα 180 μg s.c. once weekly for 48 weeks | B, 15 |
|  |  |  | **Group С:** BLV 5 mg s.c. once daily + Peg-IFNα 180 μg s.c. once weekly for 48 weeks | C, 15 |
|  |  |  | **Group D:** BLV 2 mg s.c. once daily for 48 weeks | D, 15 |
|  |  |  | **Group E:** BLV 10 mg (10 mg once daily) s.c. + Peg-IFNα 180 μg s.c. once weekly for 48 weeks | E, 15 |
|  |  |  | **Group F:** BLV 10 mg (5 mg twice daily) s.c. + TDF 300 mg orally once daily for 48 weeks, with an additional 24-week follow-up period on TDF treatment | F, 15 |

| **Ongoing clinical studies** | | | | |
| --- | --- | --- | --- | --- |
| MYR204, phase 2^b^ | France Moldova Romania Russia | 96 weeks (follow-up 48 weeks) | **Group A:** Peg-IFNα 180 μg s.c. once weekly for 48 weeks with a further follow-up period of 48 weeks | A, 24 |
|  |  |  | **Group B:** BLV 2 mg s.c. once daily + Peg-IFNα 180 μg s.c. once weekly for 48 weeks, followed by BLV 2 mg s.c. once daily for 48 weeks, with a further follow-up period of 48 weeks | B, 50 |
|  |  |  | **Group C:** BLV 10 mg s.c. once daily + Peg-IFNα 180 μg s.c. once weekly for 48 weeks, followed by BLV 10 mg s.c. once daily for 48 weeks, with a further follow-up period of 48 weeks | C, 50 |
|  |  |  | **Group D:** BLV 10 mg s.c. once daily for 96 weeks with a further follow-up period of 48 weeks | D, 50 |
| MYR301, phase 3^c^ | Germany Italy Russia Sweden | 144 weeks (follow-up 96 weeks) | **Group A:** Observation for 48 weeks followed by BLV 10 mg s.c. once daily for 96 weeks and a further follow-up period for 96 weeks | A, 51 |
|  |  |  | **Group B:** BLV 2 mg s.c. once daily for 144 weeks, with a further follow-up period of 96 weeks | B, 49 |
|  |  |  | **Group C:** BLV 10 mg s.c. once daily for 144 weeks, with a further follow-up period of 96 weeks | C, 50 |

^a^Patients included in the safety analysis set (received at least 1 dose of study drug in MYR203, MYR204, and MYR301 or were randomized to delayed treatment in study MYR301).

^b^Primary report published as Asselah T, et al. Bulevirtide combined with pegylated interferon for chronic hepatitis D. *N Engl J Med*. 2024;391:133-143. doi: 10.1056/NEJMoa2314134.

^c^Primary report published as Wedemeyer H, et al. A phase 3, randomized trial of bulevirtide in chronic hepatitis D. *N Engl J Med*. 2023;389:22-32. doi: 10.1056/NEJMoa2213429.

Abbreviations: BLV, bulevirtide; Peg-IFNα, pegylated interferon-alfa; s.c., subcutaneously; TDF, tenofovir disoproxil fumarate (as TDF 300 mg, equivalent to tenofovir disoproxil 245 mg).

| **Table S2.** Key inclusion/exclusion criteria across MYR203, MYR204, and MYR301. | | |
| --- | --- | --- |
| **Inclusion criteria** | | |
| **Study MYR203** | **Study MYR204** | **Study MYR301** |
| Male or female, aged 18-65 years (inclusive) | Male or female, aged 18-65 years (inclusive) | Male or female, aged 18-65 years (inclusive) |
| Patients with chronic hepatitis B (HbeAg-positive or negative) and HbsAg-positive for at least 6 months prior to screening. Positive for anti-HDV antibodies for at least 6 months prior to screening | Chronic HDV infection: positive serum anti-HDV antibody results or PCR results for serum/plasma HDV RNA for at least 6 months before screening | Chronic HDV infection: positive serum anti-HDV antibody results or PCR results for serum/plasma HDV RNA for at least 6 months before screening |
| Positive results for HDV RNA at screening | Positive PCR results for HDV RNA at screening | Positive PCR results for HDV RNA at screening |
| ALT level ≥1× ULN but <10× ULN | ALT level >1× ULN but <10× ULN | ALT level >1× ULN but <10× ULN |
|  | Serum albumin >28 g/L | Serum albumin >28 g/L |
| **Exclusion criteria^†^** | | |
| **Study MYR203** | **Study MYR204** | **Study MYR301** |
| Child-Pugh score of B/C or ≥6 points | Child-Pugh hepatic insufficiency score of B-C or over 6 points  *Note*: Child-Pugh hepatic insufficiency score of 6 points is allowed. Only patients with compensated cirrhosis are allowed. Uncomplicated esophageal varices allowed. Patients with current bleeding or ligation, or history of bleeding or ligation within the last 2 years are excluded | Child-Pugh hepatic insufficiency score over 7 points. Uncomplicated esophageal varices allowed; Patients with current bleeding or ligation, or history of bleeding or ligation within the last 2 years are excluded |
| HCV or HIV coinfection. Patients with HCV antibodies can be enrolled if screening HCV RNA test is negative | HCV or HIV coinfection. Patients with HCV antibodies can be enrolled if screening HCV RNA test is negative | HCV or uncontrolled HIV co-infection. Patients with HCV antibodies can be enrolled if screening HCV RNA test is negative. Patients with HIV infection can be enrolled if CD4+ cell counts are >500/mL and HIV RNA is below LOD for at least 12 months |
| A laboratory test expressing Serum creatinine >1.5× ULN | Creatinine clearance <60 mL/min as estimated using Cockcroft-Gault formula | Creatinine clearance <60 mL/min as estimated using Cockcroft-Gault formula |
| Total bilirubin >34.2 μmol/L. Patients with higher total bilirubin may be enrolled upon consultation with the study medical monitor, if there is clear evidence that the elevated bilirubin Is caused by Gilbert’s syndrome | Total bilirubin ≥34.2 μmol/L. (Patients with higher total bilirubin values may be included after the consultation with the study medical monitor, if such elevation can be clearly attributed to Gilbert’s syndrome associated with low-grade hyperbilirubinemia) | Total bilirubin ≥34.2 μmol/L. Patients with higher total bilirubin values may be included after the consultation with the study medical monitor, if such elevation can be clearly attributed to Gilbert’s syndrome associated with low-grade hyperbilirubinemia |
| Concurrent malignancy (current diagnosed or suspected malignancy; risk of a previous malignancy recurrence) | Evidence of an active or suspected malignancy, or an untreated premalignancy disorder, or a history of malignancy within the last 5 years (except for successfully treated carcinoma of the cervix in situ and successfully treated basal cell carcinoma and squamous cell carcinoma not less than 1 year prior to screening [and no more than 3 excisions of skin cancer within the last 5 years prior to screening]) or history of hepatic carcinoma | Evidence of an active or suspected malignancy or a history of malignancy, or an untreated premalignancy disorder within the last 5 years (except for successfully treated carcinoma of the cervix in situ and successfully treated basal cell carcinoma and squamous cell carcinoma not less than 1 year prior to screening [and no more than 3 excisions of skin cancer within the last 5 years prior to screening]) or history of hepatic carcinoma |
|  | Systemic connective tissue disorders | Systemic connective tissue disorders |
|  | NYHA class III-IV congestive heart failure | NYHA class III-IV congestive heart failure |
|  | Patients with uncontrolled arterial hypertension: systolic blood pressure >150 mm Hg and/or diastolic blood pressure >100 mm Hg at screening | Patients with uncontrolled arterial hypertension: systolic blood pressure >150 mm Hg and/or diastolic blood pressure >100 mm Hg at screening |
| Current or previous decompensated liver disease, including coagulopathy, hyperbilirubinemia, hepatic encephalopathy, hypoalbuminemia, ascites, and esophageal varices hemorrhage | Current or previous decompensated liver disease, including coagulopathy, hepatic encephalopathy, and esophageal varices hemorrhage | Current or previous (within last 2 years) decompensated liver disease, including coagulopathy, hepatic encephalopathy, and esophageal varices hemorrhage |
|  | Use of interferons within 6 months before screening | Use of interferons within 6 months before screening |
| WBC count <3000 cells/mm^3^ | WBC count <3000 cells/mm^3^ (<1500 if African patients) | WBC count <3000 cells/mm^3^ (<1500 if African patients) |
| Neutrophil count <1500 cells/mm^3^ | Absolute neutrophil count <1500 cells/mm^3^ (<1000 if African patients) | Neutrophil count <1500 cells/mm^3^ (<1000 if African patients) |
| Platelet count <90,000 cells/mm^3^ | Platelet count <90,000 cells/mm^3^ | Platelet count <60,000 cells/mm^3^ |
| Hemoglobin <100 g/L | Hemoglobin <12 g/dL |  |
| History of solid organ transplantation | History of solid organ transplantation | History of solid organ transplantation |
| Signs of drug and/or alcohol dependence (80 g of alcohol/day for men and 40 g of alcohol/day for women) within 1 year before screening | Current alcohol abuse or alcohol abuse within 6 months prior to enrolment in this study; current drug addict or history of drug use within 2 years prior to screening | Current alcohol abuse or alcohol abuse within 6 months prior to enrolment in this study; past or current drug addict |
| Need for concomitant use of glucocorticoids or myelotoxic agents | History of disease requiring regular use of systemic glucocorticosteroids (inhalative glucocorticosteroids are allowed) or other immunosuppressants | History of disease requiring regular use of systemic glucocorticosteroids (inhalative glucocorticosteroids are allowed) or other immunosuppressants |
| Pregnant or breast-feeding females | Pregnant or breast-feeding females | Pregnant or breast-feeding females |
| Participation in another clinical study within 30 days prior to enrolment into this study | Participation in another clinical study with investigational drugs within 30 days prior to randomization | Participation in another clinical study with investigational drugs within 30 days prior to randomization |
| Previous treatment with Myrcludex B (patients with previous exposure to interferon are eligible) | Recipient of bulevirtide previously (e.g., in clinical trials) | Recipient of bulevirtide previously (e.g., in clinical trials) |
| Antiviral therapy to treat chronic viral hepatitis B with delta-agent during the previous 6 months |  |  |
| History of immunologically mediated diseases (e.g., idiopathic thrombocytopenic purpura, lupus  erythematosus, scleroderma, severe psoriasis, rheumatoid arthritis). | History of autoimmune disorder (e.g., myositis, hepatitis, thrombotic thrombocytopenic purpura, idiopathic thrombocytopenic purpura, severe psoriasis, rheumatoid arthritis, interstitial nephritis, thyroiditis, and systemic lupus erythematosus) |  |
| Previous or current significant psychiatric disorders at screening (e.g., severe depressions, suicidal attempts, severe neuroses, or cognitive disorders) | Presence or history of significant psychiatric disorder (e.g., severe depression, suicide attempt, severe neurosis, or cognitive disorder) |  |
| Abbreviations: ALT, alanine aminotransferase; CD4, CD4 T lymphocytes; LOD, limit of detection; NYHA, New York Heart Association; ULN, upper limit normal; WBC, white blood count.  ^†^Exclusion criteria applied at the data collection or analytic stages of the study. | | |

| Table S3. Patient disposition, safety analysis set. | | | |
| --- | --- | --- | --- |
| **Screened (N = 551)** | **Control** | **BLV** | **Peg-IFNα** |
| Safety analysis set^†^ | 51 | 179 | 39 |
| Completed study (MYR203) | NA | 27 (15.1%) | 10 (25.6%) |
| Completed week 48 visit (MYR204 and MYR301) | 50 (98.0%) | 143 (79.9%) | 19 (48.7%) |
| Prematurely discontinued study | 1 (2.0%) | 9 (5.0%)^‡^ | 10 (25.6%) |
| **Reasons for premature discontinuation of study** | | | |
| Withdrawal of consent by patient | 0 | 5 (2.8%) | 4 (10.3%) |
| Adverse event | 0 | 0 | 3 (7.7%) |
| Lost to follow-up | 0 | 2 (1.1%) | 1 (2.6%) |
| Physician decision | 0 | 1 (0.6%) | 1 (2.6%) |
| Pregnancy | 1 (2.0%) | 0 | 1 (2.6%) |
| Lack of efficacy | 0 | 0 | 0 |
| Noncompliance with study drug | 0 | 1 (0.6%) | 0 |
| Other | 0 | 0 | 0 |
| Data from MYR203, MYR204, and MYR301 are included.  Percentages for completion status were calculated based on the number of patients in the safety analysis set. For studies MYR204 and MYR301, prematurely discontinued study is defined as prematurely discontinued study on or before the week 48 visit date. The control group corresponds to the MYR301 delayed-treatment group.  Abbreviations: BLV, bulevirtide; NA; not applicable; Peg-IFNα, pegylated interferon-alfa.  ^†^The safety analysis set includes all patients from studies MYR203, MYR204, and MYR301 who received at least 1 dose of study drug and 51 patients from study MYR301 who were randomized to delayed treatment.  ^‡^Three patients prematurely discontinued from the BLV 2-mg group, and 6 prematurely discontinued from the BLV 10-mg group. | | | |

| Table S4. BA levels and pruritus. | | | |
| --- | --- | --- | --- |
|  |  | **BLV 2 mg n = 64** | **BLV 10 mg n = 115** |
| **Pruritus** | Number of patients | 7 (11%) | 11 (10%) |
|  | Number of events  Serious event  Event grade 3 or 4  Event leading to D/C | 9  0  0  0 | 17  0  0  0 |
| **Time to onset, days** | Median (range) | 49 (11–305) | 97 (1–235) |
| **Duration, days** | Median (range) | 37 (1–325) | 49 (1–317) |
| **Pruritus linked to maximum BA level** | Yes/no | 1/8 | 3/14 |
| **Outcome of events** | Resolved | 9 (100%) | 15 (88%) |

Data from MYR203, MYR204, and MYR301 are included.

Abbreviations: BA, bile acids; BLV, bulevirtide; D/C, discontinuation.

| Table S5. Change in vitamin D levels from baseline to week 48. | | | | |
| --- | --- | --- | --- | --- |
|  | **Control (n = 51)** | **BLV 2 mg (n = 49)** | **BLV 10 mg (n = 100)** | **Peg-IFNα (n = 24)** |
| Baseline |  |  |  |  |
| n | 51 | 48 | 99 | 24 |
| Mean (SD), ng/mL | 25.8 (12.22) | 28.1 (12.28) | 27.8 (13.35) | 25.9 (10.19) |
| Week 24 |  |  |  |  |
| n | 49 | 48 | 94 | 16 |
| Mean (SD), ng/mL | 19.5 (11.81) | 22.6 (13.35) | 22.7 (14.63) | 22.1 (12.52) |
| Change from baseline, mean (SD), ng/mL | −6.6 (8.38) | −5.6 (8.05) ^†^ | −5.2 (9.63) ^‡^ | −6.2 (10.49) |
| Week 48 |  |  |  |  |
| n | 50 | 48 | 94 | 18 |
| Mean (SD), ng/mL | 26.8 (14.06) | 27.7 (13.72) | 29.8 (15.85) | 36.2 (14.93) |
| Change from baseline, mean (SD), ng/mL | 0.9 (10.24) | −0.2 (10.19) ^†^ | 1.8 (10.17) ^‡^ | 7.6 (11.67) |
| Data from MYR204 and MYR301 are included.  Abbreviations: BLV, bulevirtide; Peg-IFNα, pegylated interferon-alfa.  ^†^Change from baseline based on 47 patients.  ^‡^Change from baseline based on 93 patients. | | | | |

**
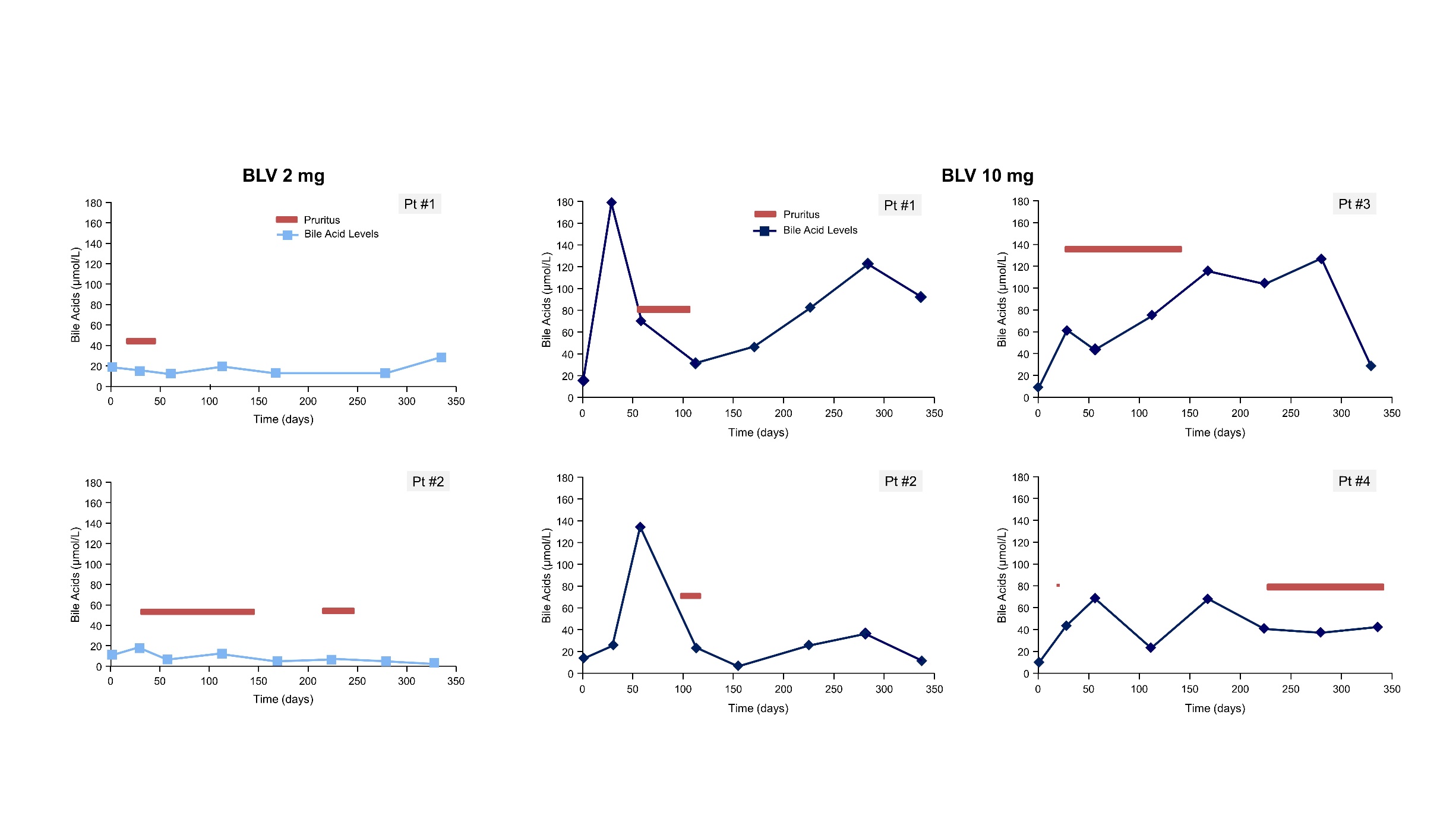
**

**Figure S1.** Selected examples of total bile acids in patients with pruritus. Data from MYR203, MYR204, and MYR301 are included. Random selection of patients shows wide intra- and interpatient BA level variability over time. Abbreviations: BA, bile acids; BLV, bulevirtide; Pt, patient.

**
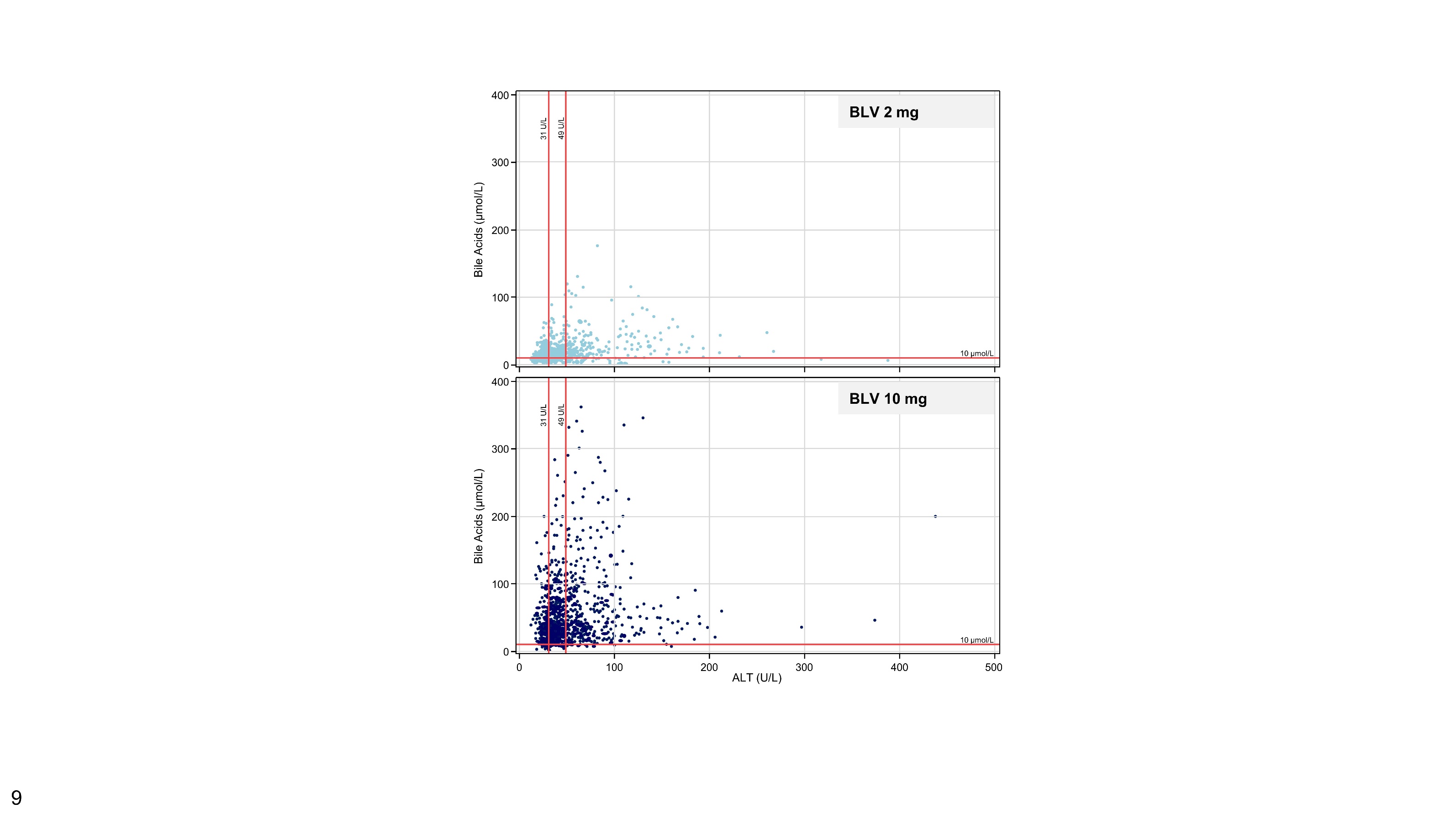
**

**Figure S2.** Relationship of total bile acid levels and ALT. Data from MYR203, MYR204, and MYR301 are included. Abbreviations: ALT, alanine aminotransferase; BLV, bulevirtide.

**
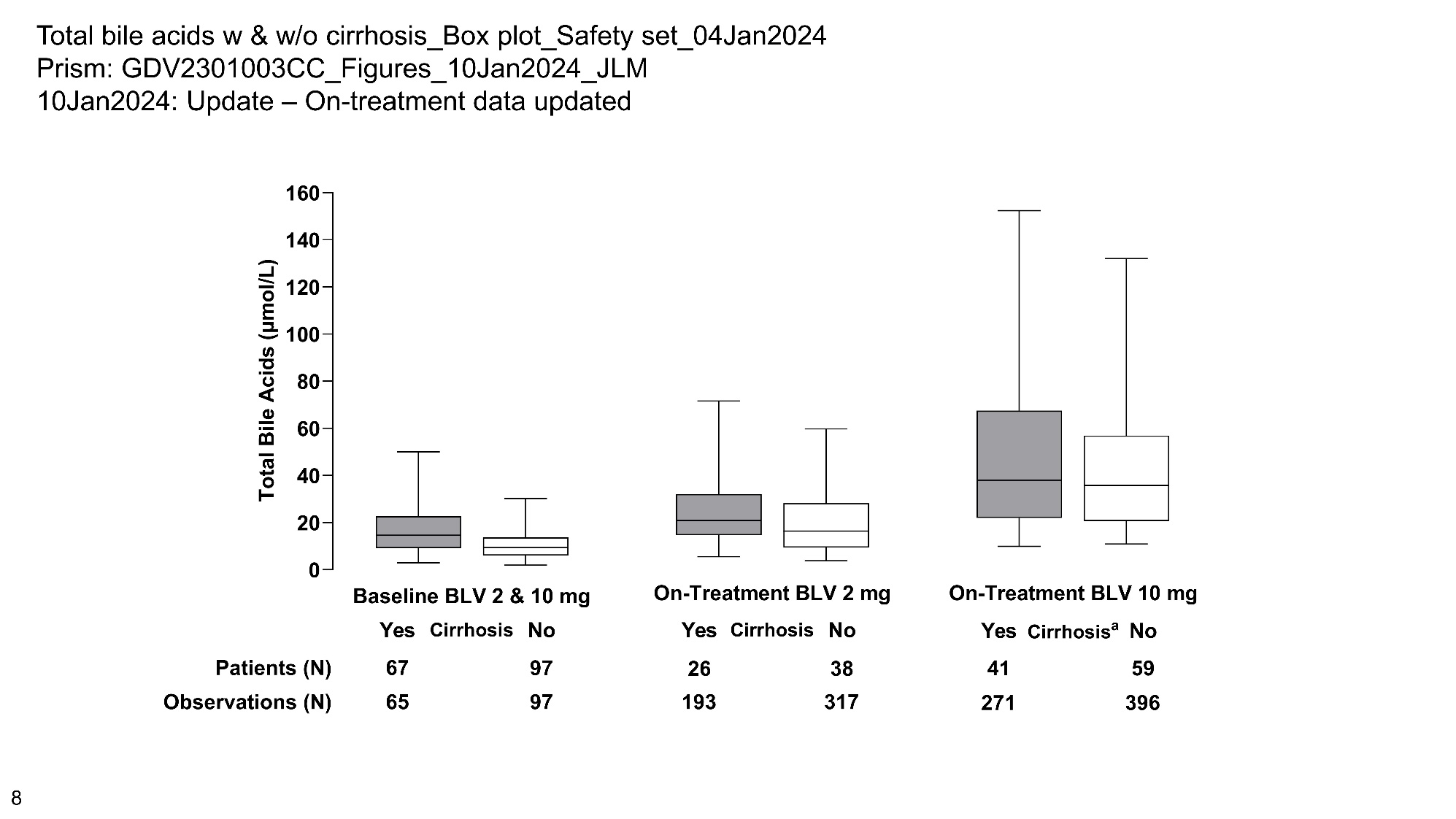
Figure S3.** Baseline vs*.* on-treatment total bile acid levels in patients with and without cirrhosis. Data from MYR203, MYR204, and MYR301 are included. Box plot horizontal lines within the shaded boxes represent the medians. The shaded boxes represent the interquartile range (25%-75%). The box plot whiskers represent the 5^th^ and 95^th^ percentile. Abbreviation: BLV, bulevirtide. ^a^Cirrhosis status was not collected for 15 patients enrolled in BLV 10 mg.
